# No evidence for induction or selection of mutant sodium channel expression in the copepod *Acartia husdsonica* challenged with the toxic dinoflagellate *Alexandrium fundyense*

**DOI:** 10.1002/ece3.1197

**Published:** 2014-08-22

**Authors:** Michael Finiguerra, David E Avery, Hans G Dam

**Affiliations:** 1 Department of Marine Sciences, University of Connecticut1080 Shennecossett Road, Groton, Connecticut, 06340-6098; 2 Department of Arts and Sciences, Maine Maritime Academy54 Pleasant Street, Castine, Maine, 04420

**Keywords:** Copepods, induction, isoform expression, selection, sodium channel, toxic algae

## Abstract

Some species in the dinoflagellate genus *Alexandrium* spp. produce a suite of neurotoxins that block sodium channels, known as paralytic shellfish toxins (PST), which have deleterious effects on grazers. Populations of the ubiquitous copepod grazer *Acartia hudsonica* that have co-occurred with toxic *Alexandrium* spp. are better adapted than naïve populations. The mechanism of adaptation is currently unknown. We hypothesized that a mutation in the sodium channel could account for the grazer adaptation. We tested two hypotheses: (1) Expression of the mutant sodium channel could be induced by exposure to toxic *Alexandrium fundyense*; (2) in the absence of induction, selection exerted by toxic *A. fundyense* would favor copepods that predominantly express the mutant isoform. In the copepod *A. hudsonica*, both isoforms are expressed in all individuals in varying proportions. Thus, in addition to comparing expression ratios of wild-type to mutant isoforms for individual copepods, we also partitioned copepods into three groups: those that predominantly express the mutant (PMI) isoform, the wild-type (PWI) isoform, or both isoforms approximately equally (EI). There were no differences in isoform expression between individuals that were fed toxic and nontoxic food after three and 6 days; induction of mutant isoform expression did not occur. Furthermore, the hypothesis that mutant isoform expression responds to toxic food was also rejected. That is, no consistent evidence showed that the wild-type to mutant isoform ratios decreased, or that the relative proportion of PMI individuals increased, due to the consumption of toxic food over four generations. However, in the selected line that was continuously exposed to toxic food sources, egg production rate increased, which suggested that adaptation occurred but was unrelated to sodium channel isoform expression.

## Introduction

The geographic expansion of many toxic marine algal species (Hallegraeff 1993; Anderson et al. 2008) raises important ecological and evolutionary considerations. Rapid evolution via natural selection to toxic algae occurs in freshwater and marine grazers such as *Daphnia* spp. (Hairston et al. 1999, 2005) and copepods (Colin and Dam 2004), respectively. This rapid evolution may mitigate the deleterious effects of toxic algae on grazers (Colin and Dam 2007), lead to polymorphisms in populations (Dam 2013), and ultimately influence population dynamics of both prey and predators (Saccheri and Hanski 2006). Hence, understanding the evolutionary relationship between toxic algae and their grazers is critical to predicting the impact of proliferating toxic algal species on aquatic food webs.

Some species in the marine dinoflagellate genus *Alexandrium* produce a suite of powerful neurotoxins (e.g., saxitoxin, neosaxitoxin, and gonyautoxin) that bind to the extracellular portion of voltage-gated sodium channels, inhibiting nerve transmissions and muscle contractions in metazoans (Cestèle and Catterall 2000; Catterall et al. 2003). Filter-feeding shellfish such as mussels, clams, and oysters can accumulate toxic cells upon ingestion of toxic algae. Human consumption of shellfish contaminated with toxic *Alexandrium* spp. can lead to paralysis and death (paralytic shellfish poisoning (Shumway 1990)); these toxins, then, are collectively known as paralytic shellfish toxins (PST).

**Figure 1:**
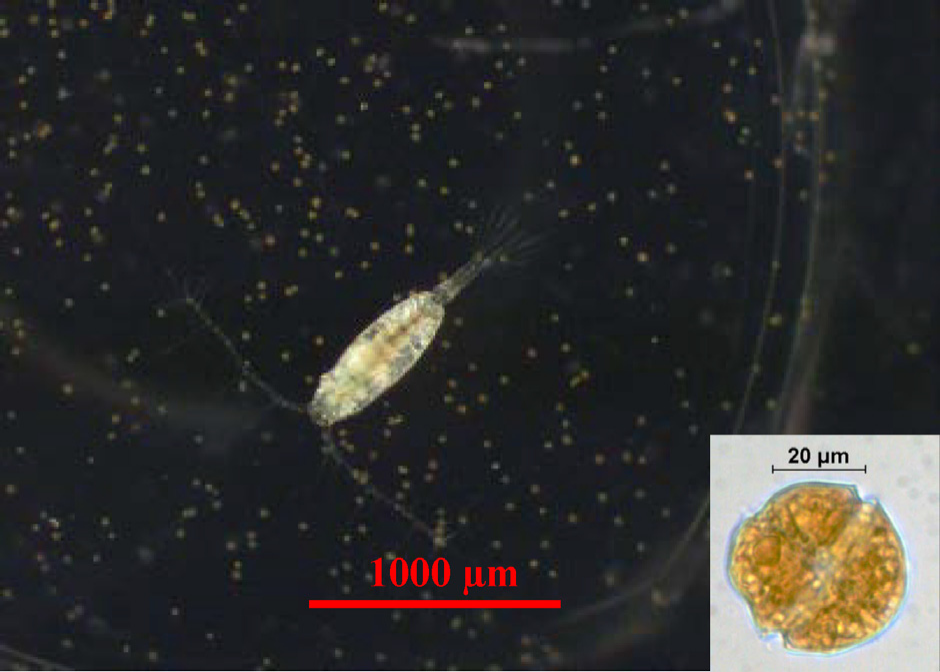
The study organisms. Main picture: The copepod *Acartia hudsonica* surrounded by PST-producing dinoflagellate prey cells, *Alexandrium fundyense*; scale bar = 1000 *μ*m, the copepod is ∼800 *μ*m. Inset: close-up of an *A. fundyense* cell; scale bar = 20 *μ*m, cell is ∼35 *μ*m.

Species of *Alexandrium* spp. that produce PST have regularly bloomed north of Cape Cod, MA, USA, (Balech 1990; Anderson et al. 1994) and more recently to the south in Long Island Sound, NY (Hattenrath et al. 2010). Copepods are primary consumers of toxic *Alexandrium* spp. and adapt to it quickly. Northern copepod populations of *Acartia hudsonica* (Fig.1) that were historically exposed to toxic *Alexandrium* spp. (e.g., Maine; exposed) performed significantly better when challenged with a diet containing toxic *Alexandrium* spp.*,* than southern populations that had not previously been exposed to it (e.g., New Jersey; naive) (Colin and Dam 2002b, 2007). Moreover, the adaptation of a naïve population of *A. hudsonica* challenged with a diet containing toxic *Alexandrium fundyense* became evident in two generations (Colin and Dam 2004). The adaptation seemed to result from a simple genetic system (Avery and Dam 2007). The mechanism that conferred this adaptation remained unknown.

Adaptation to PST and similar toxins (e.g., tetrodotoxin; TTX) involves mutations of the sodium channel. Structural changes in the sodium channel that correspond to the binding site of the saxitoxin or TTX, which are the most abundant and potent congeners and used as proxies for all PST, dramatically reduced sensitivity to the toxins, and have allowed snakes (Geffeney et al. 2002), pufferfish (Jost et al. 2008), and clams (Bricelj et al. 2005) to minimize the deleterious effects of toxic prey. Further, field mice sympatric with scorpion prey that produce toxins that also block sodium channel were found to be adapted (Rowe and Rowe 2008). Adapted field mice had mutations in specific sodium channels that blocked the pain response inflicted by toxins that the scorpions produced (Rowe et al. 2013). In all cases, mutations in sodium channels allowed grazers to perform better when ingesting toxic prey.

Clams that express the resistant sodium channels had several fitness advantages over susceptible individuals when feeding on toxic *Alexandrium* spp. Resistant genotypes had higher ingestion and survival rates, as well as increased burrowing capacities (an escape mechanism) (Bricelj et al. 2005). In geographic areas that have experienced toxic *Alexandrium* spp. blooms, juvenile soft shell clams that were homo- or heterozygous for resistance were selected for (Connell et al. 2007; Bricelj et al. 2010). To determine whether a similar mutation could account for adaptation of the copepod *Acartia hudsonica* to toxic *Alexandrium* spp. the sodium channel gene of the copepod was sequenced. Indeed, a mutant sodium channel isoform was discovered in *A. hudsonica*; however, the mutation was not at the binding site and, thus, did not alter the sensitivity of the channel to PST (Chen, 2010). That is, both wild-type and mutant sodium channels were equally susceptible to toxin binding. The mutation, however, did affect the functionality of cells in a way that may still lead to adaptation to PST.

Electrophysiological comparisons highlighted subtle but distinct differences between mutant and wild-type functioning in the sodium channel of *Acartia hudsonica* (Chen, 2010). The mutation may keep the intracellular inactivation gate from closing properly, resulting in incomplete inactivation of the channel, which potentially leads to residual currents after excitation (Chen, 2010). Similar mutations in humans lead to persistent currents in the channel and deleteriously affect cell function, leading to disease (Ashcroft 2006). Cell function is restored in such cases with sodium channel blockers (Urbani and Belluzzi 2000), which are similar in action to PSTs. Copepods that express the mutant sodium channel isoforms may be at an advantage when ingesting food that contains PSTs compared to individuals that express the wild-type sodium channel isoform.

All copepods analyzed expressed both isoforms (Finiguerra, 2013). The mutant sodium channel isoform appeared to be the result of alternative splicing in which the functional mRNA incorporated part of a noncoding intron during transcription (HG Dam Lab, unpublished data). Mutations that incorporate noncoding sequences can lead to rapid evolutionary change (Andolfatto 2005). Thus, the mutant sodium channel isoform in *Acartia hudsonica* may be a novel mechanism of adaptation to toxic *Alexandrium* spp.

In this study, we used laboratory experiments to test the hypothesis that the expression of the mutant sodium channel isoform in the copepod *Acartia hudsonica* is favored under toxic *Alexandrium* spp. conditions. First, we determined whether expression was inducible upon exposure to toxic *Alexandrium* spp. Next, we conducted a multigenerational selection experiment to test whether individuals that predominantly express the mutant isoform would be favored in toxic environments.

## Methods

### Culture of algae and copepods

Copepods for culture were collected from Avery Point, Groton, CT, USA (Latitude: 41.31519°N, Longitude: 72.06352°W**)** in January 2010 using a 200-*μ*m-mesh conical plankton net, equipped with a solid cod end, gently towed ∼1–2 m below the surface. Toxic *Alexandrium* spp. blooms have never been recorded from the sampling site; the copepod population for culturing was naïve to toxins. In the laboratory, approximately 300–400 male and female *Acartia hudsonica* were immediately separated and fed nonlimiting food rations (>600 *μ*gC L^−1^, (Besiktepe and Dam 2002)) of a nontoxic diet (standard diet) that consisted of the diatom *Thalassiosira weissfloggi* and the green flagellate *Tetraselmis sp*. in equal carbon proportions. Both food and animals were kept on a 12:12 h light:dark cycle at 15°C. The standard diet has been used routinely to rear *A. hudsonica* cultures in our laboratory (Colin and Dam 2002a,b). At least 20 generations had elapsed before running experiments, which eliminated or minimized maternal effects in populations (Falconer and MacKay 1996). For each experiment, a cohort was established by harvesting the eggs laid over a 4 day period from approximately 400 adult females; therefore, all adults in each experiment were of a similar age.

Algae cultures were maintained in triplicate semicontinuous cultures by twice-weekly dilution with F/2 nutrient medium (Guillard 1975) and maintained on a 12:12 h light:dark cycle at 18°C. The toxic dinoflagellate *Alexandrium fundyense* was originally isolated from the Bay of Fundy, NB, Canada (Colin and Dam 2002a,b). Cellular toxicities, that is, toxin content per cell, of current cultures have not changed from initial culturing in the HG Dam Laboratory; 9–16 pgSTX equivalents cell^−1^ (Colin and Dam 2002a,b), 7–14 pgSTX equivalents cell^−1^ (Senft et al. 2011), 13–26 pgSTX equivalents cell^−1^ (current study). The nontoxic congener species *Alexandrium tamarense* was provided by NOAA'S NMFS laboratory in Milford, CT (Colin and Dam 2002a,b). The toxic and nontoxic *Alexandrium* cells had similar equivalent spherical diameters (33 and 32 *μ*m, respectively) and carbon content (2.8 × 10^−3^ and 2.6 × 10^−3^ *μ*gC cell^−1^, respectively). Three days prior to their addition, one replicate of *A. fundyense* and one of *A. tamarense* were transferred to 15°C to acclimate.

### Intrageneration induction experiment

We tested whether consuming toxic *Alexandrium fundyense* could induce the expression of the mutant sodium channel isoform in *Acartia hudsonica*. A cohort of adult *A. hudsonica* was split into three treatment groups (∼300–400 adults per treatment); each group was maintained at a ration of 500 *μ*gC L^−1^ of the standard diet, with two treatment groups supplemented with 200 *μ*gC L^−1^ of toxic *A. fundyense* and the remaining treatment group supplemented with 200 *μ*gC L^−1^ of nontoxic *A. tamarense*. While some slight negative selection of toxic cells is possible (Colin and Dam 2003), animals would still ingest toxic cells at high concentrations relative to natural conditions, and toxic effects are readily evident (Colin and Dam 2002a,b). After 3 days, one of the toxic treatment groups was switched to the diet supplemented with nontoxic *A. tamarense* (toxic then nontoxic treatment, TNT), while the other two treatments were maintained on their respective toxic and nontoxic diets (TOX and NTX treatments, respectively) for three more days (experiment total: 6 days). This approach allowed us to test whether the continuous presence of toxic food was necessary for the induction of expression of the mutant sodium channel if it occurred. Forty-eight individual adult females were sampled for isoform expression from the culture prior to splitting into food treatments (initial) and again from each of the three food treatments at three (Day 3) and six (Day 6) days. For sampling, individuals were carefully captured by their antenna or urosome, to avoid crushing, with a pair of sharp-ended forceps (Dupont # 5 Tweezers), and immersed immediately into a 1.5-mL microcentrifuge tube containing 200 *μ*L Tri Reagent (MRC, Inc., Cincinnati, OH), and frozen at −80°C prior to molecular analyses (see below). Copepod mortality during the experiment was low (<10%), with no differences in mortality among treatments.

### Multigeneration selection experiment

We also tested whether sodium channel isoform expression in copepods responded to selective pressure from toxic *Alexandrium fundyense*. The experimental design was similar to Colin and Dam (2004), with differences highlighted below. Upon maturation, 48 adult female *A. hudsonica* from a large cohort (>1000 adults; reared on standard diet) were subsampled for initial isoform expression, as previously described. The cohort was then split into two groups. Each group was fed a suspension of 500 *μ*gC L^−1^ of the standard diet; one group was supplemented with an additional 200 *μ*gC L^−1^ of toxic *A. fundyense* and the other with 200 *μ*gCL^−1^ of nontoxic *A. tamarense*. Females in each group were allowed to lay eggs for 5 days. These eggs represent the first generation of selection (F1). Eggs from the F1 group that were fed nontoxic food were continuously reared on 500 *μ*gC L^−1^ standard diet + 200 *μ*gC L^−1^ nontoxic *A. tamarense* (NTX). Eggs from the group that were fed toxic food were equally separated into two additional treatments. One treatment was reared on 500 *μ*gC L^−1^ standard diet + 200 *μ*gC L^−1^ nontoxic *A. tamarense* (TNT), whereas the other was maintained on 500 *μ*gC L^−1^ standard diet + 200 *μ*gC L^−1^ toxic *A. fundyense* (TOX). Upon maturation, 48 adult female copepods from each treatment were preserved for isoform analysis, as described above. We repeated this entire procedure from the second to the fourth generation. This design allowed us to test for a response of isoform expression to continuous selective pressure (TOX line), selective pressure during the parental generations only (TNT line), and no selective pressure (NTX line).

Food suspensions for all treatment cultures were checked and refreshed every other day. The toxin content of any added *Alexandrium fundyense* was measured by HPLC analysis (Dam and Haley 2011); however, the toxicity of *A. fundyense* was not measured for the initial generation. Cultures were maintained in separate 20-L buckets. While individual treatment lines were not replicated, bucket effects were previously found to be negligible within this system (Colin and Dam 2004). Further, individual measurements of relative isoform expression and egg production rate were compared among treatments, not pooled means. Limitations of this approach are discussed below (see discussion). Samples from each treatment culture (250-300 ml) were preserved (10% formalin) twice a week, and all copepod life stages were enumerated under a dissecting microscope. Mortality rates from nauplii to adult for each generation were calculated, assuming an exponential decrease in survival with time; that is, *N*_*t*_ = *N*_*o*_ e^−*m*t^, where *m* is the mortality coefficient (day^−1^), *N*_*o*_ and *N*_*t*_ are the nauplii and adult concentrations, respectively, and the time subscripts represent the first and last days samples were preserved.

Egg production rate (EPR) on toxic and nontoxic food was measured for each treatment line at each generation. At the same time that copepods were preserved for isoform expression, 48 additional females were split into either a toxic or nontoxic food treatment, with 24 individuals per treatment. Individual copepods were fed either 500 *μ*gC L^−1^ *Tetraselmis* sp. (nontoxic treatment) or 500 *μ*gC L^−1^ toxic *Alexandrium fundyense* (toxic treatment) in 60-mm petri dishes which contained ∼45 mL of the food suspension (one copepod per dish). The toxicity of *A. fundyense* was analyzed for each EPR assay, as described above. Copepods were transferred to fresh food suspension each day, to minimize egg cannibalism, for 2 days, after which adults were immediately preserved for molecular analysis. Eggs in petri dishes were counted daily under a dissecting microscope. Thus, isoform expression and daily EPR measurements were made on the same individual. The eggs produced from these assays were disposed of after counting.

### Isoform expression analysis

Methods for isoform expression analysis were followed as previously described (Finiguerra, 2013; Zhang et al. 2013). As both sodium channel isoforms originate from one genotype (unpublished data), an RNA-based approach was needed. High-quality RNA was extracted and reverse-transcribed according to the “Modified Zymo Method” (Zhang et al. 2013). Two SYBR green (Fast SYBR, Life Technologies)-based quantitative polymerase chain reactions (qPCR), one for each isoform (primers–mutant: AscComF3 & AscMuR1; wild type: AscComF3 & AscNorR2 (Finiguerra, 2013)), were conducted for each individual copepod sample. These results were highly correlated to known isoform ratios and reproducible for the same sample; that is, with no differences among replicate wells, or replicate runs of the same sample performed on different days. Expression artifacts from long handling times were minimized because all copepods were immediately preserved after the termination of each experiment. This method allowed us to accurately calculate a ratio of wild-type to mutant isoform expression (expression ratio) for each individual copepod.

All copepods appear to express both isoforms, but in varying ratios (Finiguerra, 2013), so we used two metrics of isoform expression in our analyses. First, we treated ratios of wild-type to mutant isoforms as a continuous function. We compared values of these ratios among treatment groups. While the majority of copepods express both isoforms within a factor of 2–3 of each other, there are extremes in either direction (e.g., some individual's express relatively 100-fold more mutant isoforms compared to wild-type). These extremes could bias analyses that us the wild-type to mutant ratios. Therefore, we also partitioned copepods into expression groups: those that predominantly expressed either mutant (PMI) or wild-type isoforms (PWI), and those that expressed both channel isoforms approximately equally (EI). The PMI and PWI expression groups were defined by isoform ratios that were greater than two. To determine whether these groupings created a bias in results, we also tested other criteria for defining the expression group (e.g., using a fourfold difference for isoform ratio), but this did not alter the results, prompting us to use the above definition for all analyses. These two methods used in conjunction helped reduce bias inherent in each type of analysis.

### Statistical analyses

An ANOVA on ranks was performed when the assumptions of a parametric ANOVA were not met. Separate multiple comparisons were conducted for comparisons within treatment lines (e.g., TOX line across sampling dates) and comparisons within sampling dates among lines (e.g., F2 among three selected lines). The following comparisons failed the test of normality even after a log_10_ transformation (Shapiro–Wilk test for normality): (1) the ratios for the intrageneration induction experiments; (2) the among-generation comparison within each selected line (intergeneration selection experiment); and (3) the within-generation comparisons for the F1 and F3 generations (intergeneration selection experiment). For non-normal distributions, a Kruskal–Wallis ANOVA on ranks of the nontransformed ratios was used with Dunn's Method for a *post hoc* comparison test. The within-generation comparisons for the F2 and F4, along with the EPR data for all generations, were normally distributed, and the Holm–Sidak *post hoc* test was used to determine specific differences. Chi-square (*χ*^2^) analyses were used to determine differences among the relative proportions of expression groups within each time point and within each food treatment across time. A four-way ANOVA, with the main factors of generation, treatment line, food diet, and expression group, was performed for the EPR data of the multigeneration selection experiment. All statistical tests were conducted using Sigma Plot 11.0 or Mini Tab 16 with an alpha value of 0.05.

## Results

### Intrageneration

**Figure 2:**
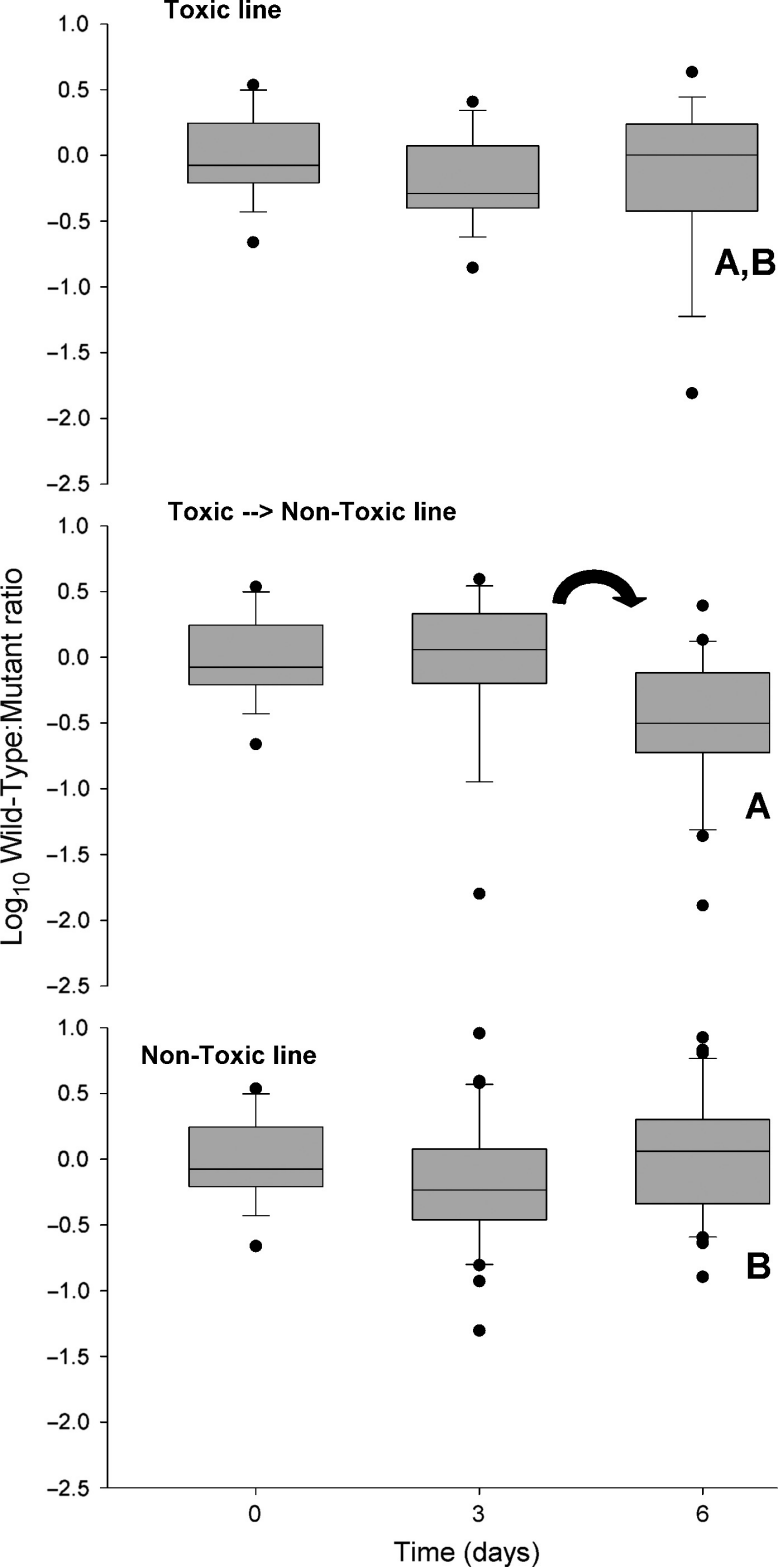
Log_10_ ratios of wild-type to mutant isoform expression in female *Acartia hudsonica* copepods exposed to toxic and nontoxic algal food over 6 days. An arrow indicates a change in ratio from the previous time point within that treatment line (ANOVA of all time points, *P* < 0.05). A letter (e.g., A, B) indicates statistical groupings (ANOVA, *P* < 0.05) among treatment lines at a given time point. Line within the box is the median; perimeter lines and whiskers are 25 & 75th and 10 & 90th percentiles, respectively; black circles are beyond the 10th percentile.

Results showed no evidence of induction of the mutant sodium channel isoform. Ratios of wild-type to mutant isoforms generally varied between −0.5 and 0.5, a less than threefold difference from one isoform to the other (Fig.2). There was no change in the ratio of wild-type to mutant isoforms for the toxic (TOX) and nontoxic (NTX) treatments over 6 days (Fig.2, ANOVA within treatments *P* > 0.05). The wild-type to mutant isoform ratio decreased from day three to day six for the toxic then nontoxic treatment (TNT; Fig.2, arrow; ANOVA, *P* < 0.05). There was no difference among treatment lines for the initial and day three sampling points (Fig.2; *P* > 0.05 ANOVA among treatment groups initial and day three). At day six, the ratio of wild-type to mutant isoforms was lower for the TNT treatment relative to the NTX treatment; however, the TOX treatment was not different than the TNT and NTX treatments (Fig.2, Day six; statistical groupings indicated by letters; *P* < 0.05 ANOVA among treatment groups).

Similarly, there was no significant change in the relative proportion of each expression group within each treatment line over the experimental period (Table1A–C; *χ*^2^ analyses, *P* > 0.05). There was no difference among treatment groups from the initial and day six time points (Table1D; *χ*^2^ *P* > 0.05). At day three, the TNT treatment had a different composition of expression groups relative to the TOX treatment; however, there was no difference between the NTX and TOX treatments (Table1 D; *χ*^2^ *P* < 0.05).

**Table 1:** Intrageneration: induction experiments – relative proportion of each sodium channel expression group for the treatments (A) fed a toxic diet for all 6 days, (B) fed a toxic diet for the first 3 days, then switched to a nontoxic diet for the remaining 3 days, and (C) fed a nontoxic diet for all 6 days. Values in parentheses represent numbers of individuals, with totals summed below. Abbreviations: equal – relatively equal expression of mutant and wild-type isoforms; mutant and wild-type – predominantly mutant or wild-type isoform expression, respectively. (D) Results of chi-square (χ^2^) analyses among treatments within each time point, letters represent statistical groupings. There were no changes in relative composition within each treatment over the 6 days. Details of statistics are found in results. The initial time point was sampled prior to separation into food treatments and is the same for each treatment. Details for definitions of expression groups are found in methods section.

	Initial	Day 3	Day 6
(A) Toxic treatment (TOX)			
∼Equal	28% (11)	43% (16)	39% (17)
Mutant	45% (18)	19% (7)	23% (10)
Wild-Type	28% (11)	38% (14)	39% (17)
Total Ind.	40	37	44
(B) Toxic → Non-Toxic treatment (TNT)			
∼Equal	28% (11)	24% (9)	18% (5)
Mutant	45% (18)	50% (19)	43% (12)
Wild-Type	28% (11)	26% (10)	39% (11)
Total Ind.	40	38	28
(C) Non-Toxic treatment (NT)			
∼Equal	28% (11)	43% (20)	24% (11)
Mutant	45% (18)	28% (13)	50% (23)
Wild-Type	28% (11)	30% (14)	26% (12)
Total Ind.	40	47	46

	Day 3	Day 6
(D) Comparisons among treatments within time point		
TOX	A	–
TNT	B	–
NT	A, B	–

### Multigeneration selection experiment

The daily mortality rate from nauplii to adult was low and comparable among treatment lines for the first two generations, with adult population densities between 350–400 L^−1^, or approximately 3500–4000 total individuals per treatment vessel. There was an overall decrease in adult population density from the second to the fourth generation but each treatment line typically maintained populations greater than 2000 individuals per incubation vessel. There was an approximate twofold increase in mortality rate during the third generation for the TOX treatment, with a subsequent reduction in adult population density to ∼50 L^−1^, or 500 adults per container. The NTX treatment line experienced a similar, but less severe, increase in mortality rate during the fourth generation. The toxicity of *A. fundyense* used for the TOX and TNT treatments ranged from approximately 12–20 pgSTX equivalents cell^−1^, with no statistical change over the four generations. There were no visible parasites, egg predators, or signs of disease affecting the copepods in any culture containers during the entire experimental period.

**Figure 3:**
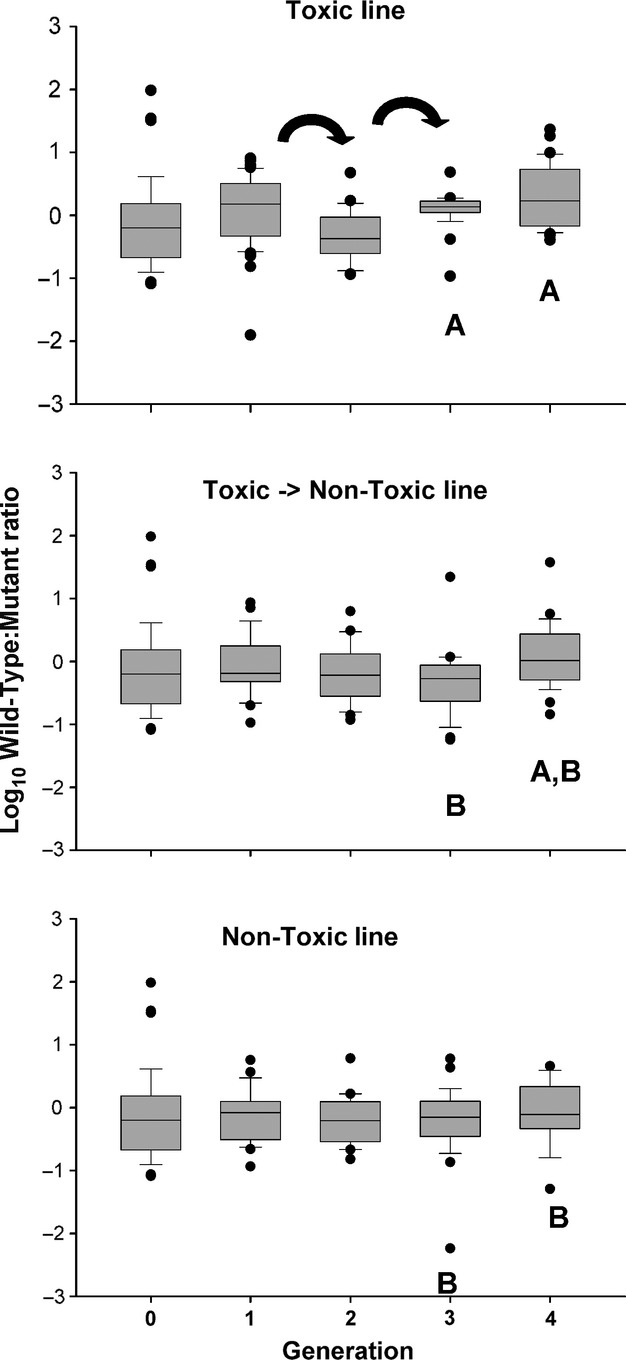
Log_10_ ratios of wild-type to mutant isoform expression in female *Acartia hudsonica* copepods over four generations of exposure to toxic *Alexandrium fundyense*. Arrows represent a change in ratio from the previous generation within each selected line (ANOVA results from all time points, *P* < 0.05), and letters (e.g., A, B) represent statistical groupings among selected lines within each generation (ANOVA, *P* < 0.05). Line within the box is the median; perimeter lines and whiskers are 25 & 75th and 10th & 90th percentiles, respectively; black circles are beyond the 10th percentile.

Comparisons of wild-type to mutant isoform ratios within each treatment line showed little evidence for selection of the mutant isoform in toxic environments. The TOX line saw the only change during the four generations: The ratio decreased after the first generation, then increased after the second (Fig.3, arrows; ANOVA within each line, *P* < 0.05). There were no differences among lines within the initial, first, and second generations. The TOX treatment line had a higher wild-type to mutant isoform ratio than the TNT and NTX treatment lines during the third generation (Fig.3, generation three; letters for statistical groupings: ANOVA, *P* < 0.05). During the fourth generation, the NTX line had a lower ratio than the TOX line; however, each was equivalent to the TNT line (Fig.3, letters for statistical groupings: generation four; ANOVA, *P* < 0.05, Kruskal–Wallis post hoc test).

Differences among the relative proportions of sodium channel expression groups also provided no support for the selection hypothesis. There was a change in proportions of expression groups for the TOX line from the F2 to F3 and F3 to F4 generations (Table2A; arrows, *χ*^2^, *P* < 0.05). The TNT line changed between the F0 and F1, and again from F2 to F3 and F3 to F4 (Table2B, arrows; *χ*^2^, *P* < 0.05). In contrast, the NTX line only changed from the F0 to the F1 (Table2C, arrows; *χ*^2^, *P* < 0.05). Differences among lines at each generation were not consistent across generations. For the F1, TOX was different than both TNT and NT lines, which were the same as each other (Table2D; *χ*^2^, *P* < 0.05). There were no differences during the F2 generation, yet all treatment lines differed from each other during the F3 generation. The TOX and TNT lines were the same for the F4 generation; however, TNT and NT were as well (Table2 D; *χ*^2^, *P* < 0.05).

**Table 2:** Intergeneration: selection experiments – relative proportion of each sodium channel expression group for selected lines (A) fed a toxic diet continuously, (B) fed a toxic diet during the parental generation, with offspring reared on a nontoxic diet, and (C) fed a nontoxic diet continuously. Values and abbreviations are the same as Table1. Arrows between generations indicate a change in composition from the previous generation. (D) Results of chi-square (χ^2^) analyses among treatments within each time point, letters represent statistical groupings. Details of statistics are found in results. The F0 time point was sampled prior to selection and is the same for each treatment line. Details for definitions of expression groups are found in methods section.

	F0	F1	F2 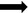	F3 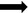	F4
(A) Toxic treatment (TOX)					
∼Equal	33% (15)	42% (20)	35% (15)	14% (6)	43% (20)
Mutant	39% (18)	29% (14)	49% (21)	26% (11)	9% (4)
Wild-Type	28% (13)	29% (14)	16% (7)	60% (25)	49% (23)
Total Ind.	46	48	43	42	47

	F0 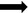	F1	F2 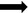	F3 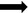	F4
(B) Toxic → Non-Toxic treatment (TNT)					
∼Equal	33% (15)	13% (5)	27% (12)	56% (25)	38% (16)
Mutant	39% (18)	35% (14)	36% (16)	33% (15)	21% (9)
Wild-Type	28% (13)	53% (21)	36% (16)	11% (5)	40% (17)
Total Ind.	46	40	44	45	42

	F0 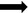	F1	F2	F3	F4
(C) Non-Toxic treatment (NT)					
∼ Equal	33% (15)	14% (6)	47% (20)	35% (16)	29% (12)
Mutant	39% (18)	42% (18)	40% (17)	33% (15)	39% (16)
Wild-Type	28% (13)	44% (19)	14% (6)	33% (15)	32% (13)
Total Ind.	46	43	43	46	41

	F1	F2	F3	F4
(D) Comparisons among treatments within time point				
TOX	A	–	A	A
TNT	B	–	B	A, B
NT	B	–	C	B

**Figure 4:**
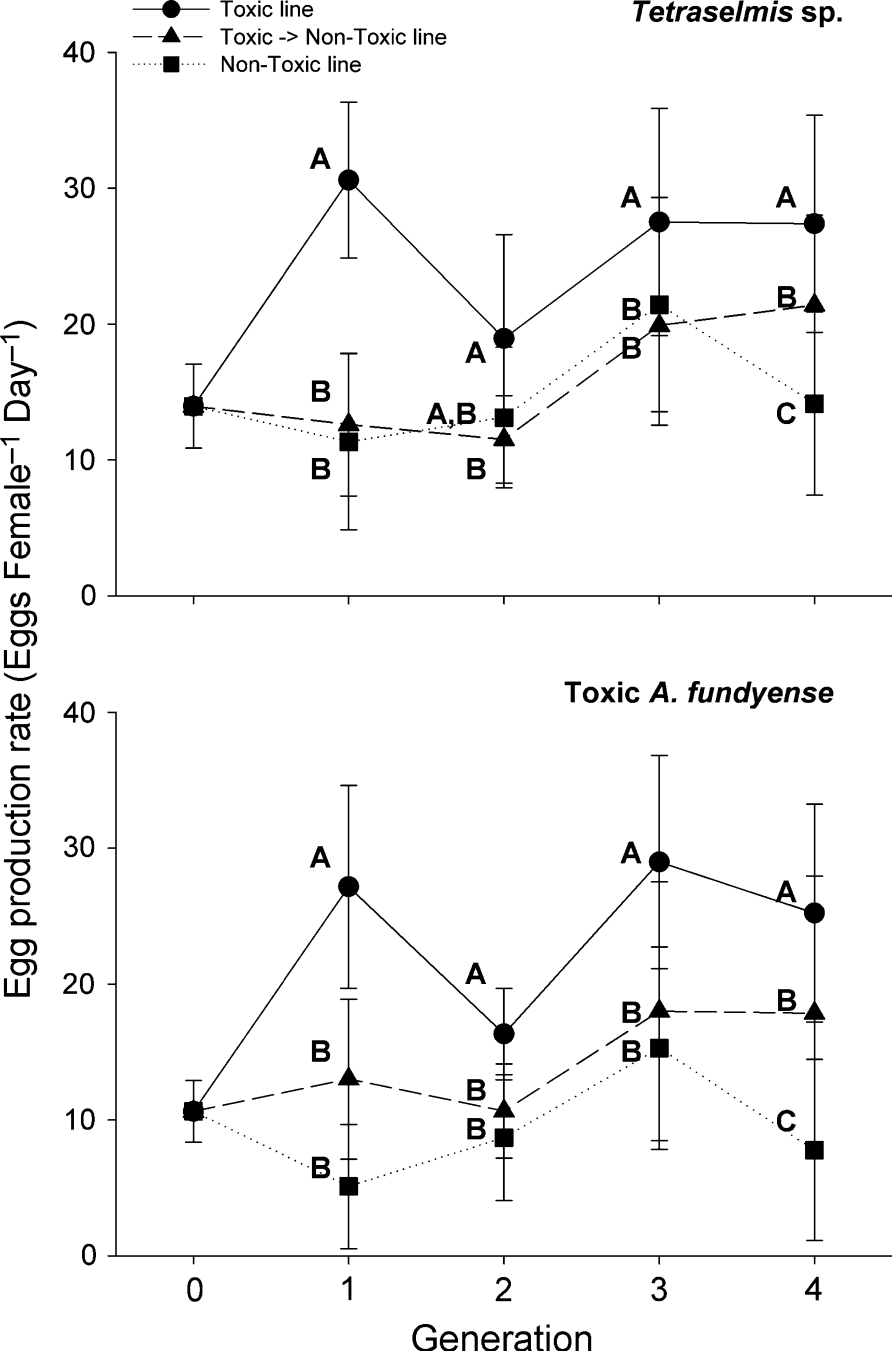
Egg production rates of individual *Acartia hudsonica* copepods from each treatment line fed nonlimiting rations of nontoxic *Tetraselmis sp*. (top panel) or toxic *Alexandrium fundyense* (bottom panel). The sample size was ∼24 individuals for each data point. Letters to the left of each data point represent statistical groupings among selected lines within each generation (ANOVA, *P* < 0.05). Error bars represent ± 1 standard deviation.

Individual egg production rates (EPR) of copepods showed rapid changes in the continuous presence of toxic algae. The TOX line had the highest EPR on both nontoxic (*Tetraselmis* sp.) and toxic (*Alexandrium fundyense*) food for all generations, except during generation two on toxic food for which TOX EPR did not differ from the TNT line (Fig.4, letters; ANOVA, *P* < 0.05 among lines at each generation). Changes in EPR between successive generations were similar between food treatments (Table3). Generally, the TOX treatment changed among generations, but remained high, while the TNT and NTX treatment lines had little change in EPR from one generation to the next (Table3, ANOVA, *P* < 0.05). The toxicity of *A. fundyense* used for EPR assays varied among generations (F0: 19.2 ± 1.2; F1: 15.1 ± 0.8; F2: 13.1 ± 1.2; F3: 26.5 ± 1.4; F4: 18.8 ± 0.4 pgSTX equivalents cell^−1^), but was not associated with any concurrent changes in EPR. A two-fold increase in cell toxicity in the F3 generation corresponded to an EPR that was actually higher from the previous generation (Table3B, ANOVA, *P* < 0.05). Results of the four-way ANOVA showed a significant main effect for generation, treatment line, and food diet, and an interaction effect between generation and treatment line (Table4, *P* < 0.01). These differences did not appear to be related to the sodium channel expression groups because no interaction effect was observed when all factors were considered (Table4; *P* > 0.05, Generation × Line × Diet × Exp group).

**Table 3:** Change in egg production rate of individual *Acartia hudsonica* copepods in successive generations within treatment lines (*n* = ∼24 per food treatment for each treatment line). An upward facing arrow indicates an increase in EPR within that treatment from the previous generation a downward facing arrow a decrease in EPR (*t*-test, *P* < 0.05). A dash indicates no change from the previous generation (*t*-test, *P* > 0.05).

Generation of selection				
F0	F1	F2	F3	F4
(A). Non-Toxic *Tetraselmis sp*.				
TOX	↑	↓	↑	–
TNT	–	–	↑	–
NTX	–	–	↑	–
(B) Toxic *Alexandrium fundyense*				
TOX	↑	↓	↑	–
TNT	–	–	↑	–
NTX	–	–	↑	↓

**Table 4:** Four-factor ANOVA comparing the effects of generation (Gen), treatment line (Line), nontoxic or toxic diet (Diet), and expression group (Exp group) on egg production rate of *Acartia hudsonica* copepods during the multigeneration selection experiment.

Source	DF	Seq SS	Adj SS	Adj MS	*F*-value	*P*-value
Gen	3	6416	4432	1477.4	35.4	<0.001
Line	2	10634	7436	3717.9	89.08	<0.001
Diet	1	415	374	374.4	8.97	<0.01
Exp group	2	50	53	26.3	0.63	0.53
Gen × Line	6	3998	2868	477.9	11.45	<0.001
Gen × Diet	3	92	63	21.1	0.51	0.68
Gen × Exp group	6	363	286	47.7	1.14	0.34
Line × Diet	2	47	16	7.8	0.19	0.83
Line × Exp group	4	96	82	20.4	0.49	0.74
Diet × Exp group	2	42	4	2.2	0.05	0.95
Gen × Line × Diet	6	411	287	47.9	1.15	0.33
Gen × Line × Exp group	12	227	212	17.7	0.42	0.95
Gen × Diet × Exp group	6	31	48	8.0	0.19	0.98
Line × Diet × Exp group	4	52	75	18.9	0.45	0.77
Gen × Line × Diet × Exp group	12	504	504	42.0	1.01	0.44
Error	405	16,904	16,904	41.7		
Total	476	40,281				

## Discussion

The ability of other organisms to adapt to neurotoxic prey resulted from mutations in the sodium channel. A recently discovered mutant sodium channel isoform in the copepod *Acartia hudsonica* was hypothesized to be the mechanism of adaptation to the toxic algal *Alexandrium fundyense*. Our results provide little support for the hypothesis that expression of the mutant sodium channel isoform is adaptive to PST-containing prey, either through induction or selection. Other novel mechanisms that confer an adaptive advantage to copepods challenged with PSTs must exist.

### Induction of mutant sodium channel isoforms

Inducible defenses allow an organism to mitigate damage in subsequent encounters with a stressor (Harvell 1990) and are common in aquatic predator–prey interactions (Tollrian and Harvell 1999). Some acquired traits, including inducible defense, are heritable (Richards 2006; Bossdorf et al. 2008). For example, tolerance to the toxic cyanobacteria *Microcytsis* sp. was developed within the life span of exposed *Daphnia magna* and passed on to offspring (Gustafsson et al. 2005). Furthermore, alternative splicing of genes, which we suspect controls mutant isoform expression, can be regulated during an organism's life span, This regulatory change can potentially be passed on to offspring (Luco et al. 2010); rapid evolution could ensue from these induced variations in fitness-related traits (Rasanen and Kruuk 2007; Bossdorf et al. 2008; Jiang et al. 2011). Therefore, we hypothesized that expression of the mutant sodium channel isoform of *Acartia hudsonica* could be induced upon exposure to PST-producing prey, and, if so, that this induction was heritable.

The induction hypothesis was tested in several ways. As measuring isoform expression is a destructive process, we used a comparative approach. Induction assumes that isoform expression is not fixed, but sensitive to regulation from external factors (e.g., PST). We also assumed that all copepods' potential for expression was similar. We did not consider a longer period of induction because the typical life span for adult copepods in the wild is a few days (Peterson 2001). Overall, there was no evidence to support our induction hypothesis. That is, a toxic food diet did not decrease the wild-type to mutant isoform ratio, or increase the relative contribution of PMI individuals. The experiments were designed to test whether induction of mutant isoforms required the continuous presence of toxic *Alexandrium fundyense*. Specifically, the TNT and TOX treatments should have differed after 6 days. Similarly, for the multigeneration experiment, the TNT line should have differed from the TOX line. Differences among these treatment lines were contradictory to predictions and inconsistent across experiments. Without evidence for induction, the heritability of induced changes was not tested. All indications are that sodium channel isoform expression was not inducible within 6 days of toxin exposure. If the mutant sodium channel isoform was responsible for adaptation in *Acartia hudsonica*, then it would be from the selection of individuals that predominantly express mutant isoforms.

### Selection of individuals that express mutant sodium channel isoforms

An alternative hypothesis to induction predicts that exposure to PST-producing algae would select for individuals that predominantly express mutant isoforms. Such an outcome could explain the adaptation of populations of the copepod *Acartia hudsonica* that were historically exposed to toxic *Alexandrium* spp. algae (Colin and Dam [Bibr b13][Bibr b14], 2004, 2007) and the rapid adaptation of naïve copepod populations (Colin and Dam 2004). While the experimental design does not unequivocally rule out the selection hypothesis, there was little evidence to support a response to selection.

The multigeneration selection experiment monitored sodium channel isoform expression and egg production rate (EPR), a metric of fitness, of individuals. Importantly, treatment cultures were maintained in individual vessels, with no replication. While culture vessel effects were not found using a similar experimental design (Colin and Dam 2004), experimental variability still could have influenced the results presented here. For example, the TOX line suffered high mortality from nauplii to adult during generation three compared to previous generations as well as the other treatment lines. There was no immediately apparent cause to this isolated mortality event (e.g., no parasites, deficient water quality, etc.); however, we cannot definitively say this mortality event did not influence results. Thus, while there is no consistent support of a response to selection, the possibility may exist and caution must be taken in interpreting the results.

Exposure of the parental lineage was shown to maintain adaptation to PSTs even in the absence of toxic food during rearing (Colin and Dam 2004). The TOX and TNT should have both had increased mutant isoform expression compared to the NTX line. This was not observed for the ratio of wild-type to mutant isoforms (Fig.3) and the relative proportion of each expression group (Table2). More broadly, the TOX treatment line was expected to have changed toward greater expression of the mutant isoform, compared to the NTX line, over the four generations. The opposite was observed; the TOX treatment had greater proportion of PWI individuals (Table2). It is not likely that the wild-type isoform responded to PST exposure. We did not observe the successive enrichment in PWI individuals in the TOX treatment over four generations. Similar changes in the relative contribution of expression groups were also observed for the TNT line, independent of the continuous presence of toxic food. Finally, there was relatively little change in the toxicity of *Alexandrium fundyense* to explain the fluctuating isoform changes.

A lack of interactive effects is also inconsistent with the selection hypothesis. Expression group was the only main factor to have no effect on EPR, with Generation x Treatment Line as the only significant interaction effect (Table4). This implies that selection occurred and was related to the food treatment but not the sodium channel expression groups. This inference was supported by the increased EPR of the TOX line over successive generations and compared to the other treatment lines. Evidence of evolutionary adaption in the present study is consistent with previous work (Colin and Dam 2004), but the sodium channel expression groups were not associated with such selection (Table4, no interaction effect with expression groups).

Our study's experimental design, including the three treatment lines, is similar to that of Colin and Dam (2004), with two important exceptions, which preclude direct comparisons of EPR. Whereas Colin and Dam (2004) measured EPR on a toxic diet for pooled samples (*n* ≈ 10 female copepods per bottle) in the NTX and TNT treatments only, we measured individual EPR on toxic and nontoxic food for all treatment lines. Our experimental design yielded the interesting observation that egg production rates for the TOX treatment line were routinely higher on both toxic and nontoxic food compared to individuals from the initial generation (F0) and the NTX and TNT treatments (Fig.4); something Colin and Dam (2004) did not measure and could not test. The increased EPR could explain the similar population sizes among all three treatments, even though the total amount of eggs from the parental toxin line was split in half to create the TOX and TNT line for the next generation, while all eggs were used for each successive NTX line (see methods). There was no evidence that directional selection in the TOX line for individuals tolerant to toxic *Alexandrium* spp. occurred because there was no concurrent increase in EPR for the TNT line.

Acute exposure (e.g., 1–2 days) to toxic cells typically reduced feeding and reproduction rates in copepods (Turner and Tester 1997; Turner 2006). The long-term effects (e.g., 3–7 days) of *Alexandrium* spp. toxins on copepod performance remain relatively unexplored. When switched from a toxic to nontoxic diet after 6 days, the egg production rate of the copepod *Acartia clausii* appeared to increase; however, it was unclear whether this was a significant difference or not (Dutz 1998). The mechanism that would allow for an increase in EPR after prolonged exposure to toxic food is unknown. It may be related to lingering effects of toxin, which putatively decreases gut passage time and may increase assimilation efficiency. This would allow a potential increase in EPR, even when fed toxic food. This hypothesis requires further testing. Alternatively, prolonged exposure to toxic food may have caused an acclimation response. Phenotypic plasticity of this nature would not be seen in the absence of toxic food during rearing (e.g., TNT line of selection experiments), nor in short one to 3-day incubations commonly used by investigators. Overall, the results of this experiment could suggest that, under prolonged toxic blooms in nature, the fitness of certain copepods may increase; however, this idea requires more rigorous testing and merits further attention.

In the absence of a clear link between the sodium channel mutation and adaptation to PST, other mechanisms of adaptation must be present. Selection for individuals that mitigate the effects of toxins may contribute to adaptation. The production of proteins that bind to PSTs and keep them from blocking sodium channels, such as the saxiphilins (Morabito and Moczydlowski 1994; Lenarčič et al. 2000; Yotsu-Yamashita et al. 2001), has not been investigated in *Acartia hudsonica,* but saxiphilin production has been observed in other crustaceans (Llewellyn et al. 1997; Llewellyn 2006) and deserves attention. At the cellular level, there are other ion channels that are susceptible to PSTs such as nonvoltage-gated sodium channels (Liebeskind et al. 2012) and calcium channels (Llewellyn, 2009). While mutations in these channels, if they exist, could contribute to adaptation to PSTs, their roles in copepod physiology are unknown.
